# The handheld fan for chronic breathlessness: Clinicians’ experiences and views of implementation in clinical practice

**DOI:** 10.1371/journal.pone.0294748

**Published:** 2023-11-28

**Authors:** Joshua Brown, Isobel Miller, Matilda Barnes-Harris, Miriam J. Johnson, Mark Pearson, Tim Luckett, Flavia Swan

**Affiliations:** 1 Wolfson Palliative Care Research Centre, Hull York Medical School, University of Hull, Kingston Upon Hull, United Kingdom; 2 Faculty of Health, Improving Palliative, Aged and Chronic Care through Clinical Research and Translation, University of Technology Sydney, Sydney, Australia; Kaohsiung Medical University Hospital, Kaohsiung Medical University, TAIWAN

## Abstract

**Introduction:**

The handheld fan (‘fan’) is useful for chronic breathlessness management, however little is known about clinicians’ implementation of the fan in clinical practice.

**Aim:**

To explore clinicians’ experiences and views of fan implementation.

**Methods:**

A qualitative approach, using semi-structured interviews. Participants were purposively sampled from clinicians who had completed an on-line fan implementation survey and were willing to participate. A topic guide was developed using the Theoretical Domains Framework (TDF). Data were analysed using an inductive approach informed by the TDF.

**Findings:**

Twelve clinicians participated (doctors n = 4; nurses n = 4; allied health professionals n = 4) from respiratory and palliative care. Analysis generated three major themes: i) Clinician knowledge and skills in fan implementation, ii) environmental constraints on fan use and iii) clinician beliefs about the consequences of fan use.

Implementation by clinicians was positively influenced by having a scientific rationale for fan use presented (mechanism of action). Clinicians believed that the fan relieved breathlessness and did not carry a significant infection risk. Opportunity for fan use varied across healthcare settings; key environmental influences were COVID-19 restrictions, lack of access to resources and funding to provide fans, particularly in acute and respiratory services. Clinicians commonly encountered scepticism among patients and colleagues who felt the fan was an implausible intervention for breathlessness.

**Conclusion:**

Implementation of the fan is motivated by clinician beliefs about patient-benefit, a scientific rationale to counter clinician and patient scepticism, and access to fans in clinic. Funding to allow patients to be supplied with and taught how to use a fan would support uptake. Research is needed to address concerns about infection risk.

## Introduction

People with progressive conditions such as cancer, heart failure and Chronic Obstructive Pulmonary Disease (COPD) frequently experience disabling chronic breathlessness [1]. Pathways for the perception of breathlessness, and the emotional response to it are well delineated [2]. Holistic services are endorsed for effective clinical management of chronic breathlessness [3–5] and incorporate non-pharmacological measures targeting such breathlessness perception pathways such as cool facial airflow delivered from a handheld battery-operated fan (‘fan’), in addition to optimal disease-directed management. Cool airflow is thought to work by modulation of central perception of breathlessness through stimulation of the trigeminal nerve, nasal mucosa and upper airway flow receptors, all acting to decrease neural respiratory drive [6–11].

Evidence supports airflow from a fan as an effective non-pharmacological intervention for relief of this debilitating symptom in cancer and chronic respiratory conditions [12–15].

The fan is recommended in guidelines for breathlessness management in cancer [16], palliative care [17] and is a component of a crisis plan for acute on chronic breathlessness from the American Thoracic Society [18].

In addition, the fan offers important benefits for self- management of breathlessness. A multi-methods secondary analysis of qualitative interview data from patient-participants in three trials of the fan in people with progressive cardiorespiratory conditions such as COPD found that the majority, over 80% experienced benefit [19] and a secondary pooled analysis of two trials of the fan in people with chronic breathlessness found over 50% reported increased physical activity [20].

Patients identified the shorter recovery time from exertional breathlessness, the fan’s portability, low cost and ease of use as important facilitators of breathlessness self-management [21–23] However patients have also highlighted that the fan may not always be perceived as a valid intervention for breathlessness [24] and technical problems exist with operability, robustness and noise [19], as well as individual preferences for different airflow speeds [19,25]. These findings emphasize the importance of the way the fan is implemented with patients [26] but little is known about how clinicians’ implement the fan in clinical practice. A scoping review explored patient, carer and clinician implementation of non-medical aids for chronic breathlessness management found no studies focussing on implementation from the clinicians’ viewpoint [27]. Since then, one study of Australian specialist respiratory clinicians’ fan use with COPD patients has been published [28]. This found that a lack of clarity about whose role it was to implement the fan, what advice to provide patients, and limited access to fans in hospitals were barriers to use [28].

We aimed to explore whether similar issues exist in the UK, and whether the COVID-19 pandemic guidance for clinicians (do not use a fan for breathlessness due to suspected risk of infection) [29,30] has added a further barrier to current fan implementation in clinical practice.

## Methods

This was a qualitative in-depth semi-structured interview study. Ethical approval for the study was granted by HYMS Faculty Ethics Committee, University of Hull July 2020 (REF 20–28). The study is reported in accordance with the Consolidated criteria for Reporting Qualitative studies (COREQ) [31].

Eligible participants were clinicians of any discipline involved in the care of people with chronic breathlessness in any healthcare setting. Clinicians were invited to leave their contact details (telephone and/or email address) if they were willing to be considered for an individual telephone interview following completion of a short on-line survey about fan implementation and the barriers to implementation. This paper reports the qualitative interview findings, and presents an in-depth exploration of clinician’s views and experience of fan use (or not) in clinical practice. The fan survey results and a mixed method data synthesis will be presented elsewhere.

### Sampling and recruitment to the interview study

Participants were purposively sampled from the list of clinicians who were willing to complete an interview using a sampling framework to maximise variation that included fan implementation (Yes/No), multi-disciplinary team (MDT) members; doctors, nurses, and allied health professionals (e.g., physiotherapists, occupational therapists), and healthcare setting such as primary, secondary and Specialist Palliative Care Unit (SPCU) e.g., hospice.

Clinicians who provided their contact details were able to download the participant information sheet and consent form (See S1 File) or received them by email according to their preference. Participants were recruited to the study from November 2020 to June 2021.The researcher (FS) telephoned potential interview participants to discuss the study, answer any questions and check eligibility in relation to the sampling framework. If eligible to complete an interview, a time and date for a telephone interview was agreed. Verbal consent was recorded at the start of their interview prior to data collection using an approved consent script. (See S1 File).

### Sample size

We anticipated a sample size of up to 20 participants would provide sufficient information power given the narrow topic focus [32]. The topic focus was restricted to healthcare professionals with anticipated differences between palliative care and non-palliative clinical specialities assessed from previous researcher fan experience and published data to date on fan implementation [28]. The need for further interviews was reviewed and we specified the stopping criterion following two interviews without any new codes in the data [33].

### Data collection

A topic guide was developed from the existing literature and criteria from the theoretical domains framework (TDFv2) [34,35], whilst allowing for unanticipated issues to be presented. This was piloted prior to use (see S2 File). Interviews were conducted by FS (a female, postdoctoral researcher with a physiotherapy clinical background and previous qualitative interview experience) by telephone and audio-recorded. FS completed anonymised field notes following each interview to document the interviewee tone (e.g., passionate, frustrated) and expression of their voice in relation to what they were saying about fan implementation during the telephone interview.

Verbatim transcription and anonymisation of the interviews were undertaken by two researchers, JB and IM, therefore none of the research team had access to information that could identify participants after data collection.

### Analysis

The anonymised transcripts were imported into NVivo Version 12 (QSR international) software [36]. The data were analysed using an inductive approach, applying the TDFv2 following guidance from Atkins et al [35]. Thematic analysis [37] was used to structure and categorise according to the relevant key domains of the TDFv2 [34,35] whilst also allowing for coding of unexpected concepts. This followed a process of immersion in data followed by line-by-line coding. Four transcripts were initially independently coded by the researchers, FS, JB, IM and MBH to agree a codebook, after which all transcripts were coded by JB and IM with support from FS. Codes were first grouped into initial themes, then into more analytic themes and sub-themes through checking and discussion with the whole team. These were then mapped against the domains and constructs of the TDFv2. Interpretation used a modified grounded theory perspective [38] as the data were approached with specific research questions in relation to clinician implementation of the fan. Participants were not sent their transcripts for checking and did not contribute to interpretation.

## Results

### Demographics

Thirteen interviews lasting between 30 to 40 minutes were conducted between November 2020 and July 2021. One interview was excluded due to the poor audio quality which prevented transcription. Data were more similar than expected therefore data saturation was achieved when no further codes arose, and further recruitment was stopped.

Participants (male = 2; female = 10) represented a wide range of disciplines (doctors, nurses and Allied Health Professionals), specialities (palliative care, respiratory and emergency medicine) and work settings (community, hospital and specialist palliative care units)as delineated in the sampling frame (see Table 1). Of note, all clinicians were implementing the fan apart from one.

**Table 1:** Characteristics of clinicians participating in interviews on the fan for breathlessness management (N = 12).

Characteristic	N
Clinician’s role	Doctors n = 4 Nurses n = 4 including Advanced Clinical Practitioner Allied Health professionals n = 4 including Physiotherapist, Occupational Therapist and Paramedic
Gender	Male = 2 Female = 10
Clinician Fan Implementation	Yes = 11 No = 1
Work setting	Community n = 2 Secondary care n = 5 Special Palliative Care Unit n = 1 Community and secondary care n = 3 Community, secondary and SPCU n = 1
Clinician speciality	Palliative care n = 6 Respiratory n = 5 Emergency care n = 1

### Findings

Three key themes were generated from the data and were coded to the domains of the TDF in relation to the clinician’s capability, motivation and opportunity to implement the fan (see Table 2 themes, sub-themes and TDF domains). Key TDF domains were: knowledge, skills; environmental context and resources, and beliefs about consequences.

**Table 2:** Themes, sub-themes and theoretical domains framework domains.

Theme	Subtheme	COM-B	TDF domains	TDF Component constructs
1. Clinician knowledge and skills of fan implementation	a) Explanation and scientific rationale b) Complex intervention c) Knowledge of fan research and research champions	Capability	Knowledge Skills	Knowledge of intervention and condition Scientific rationale Cognitive skills and ability Competence Research knowledge
2. Environmental constraints on fan use	a) Lack of access and funding for fan resources (respiratory *versus* palliative care setting) b) COVID-19 restrictions on fan use (acute *versus* community setting) c) Clinicians’ awareness of fan (generalist *versus* specialist setting)	Opportunity	Environmental context and resources	Resources; funding and access Organisational culture/climate with COVD-19 Barriers and facilitators circumstances of clinicians
3. Clinician beliefs about consequences of fan use	a) Benefit from fan use b) Low infection risk (COVID-19) from fan use. c) Patient and clinician beliefs about fan credibility as an intervention for breathlessness management	Motivation	Beliefs about consequences	Beliefs Outcome expectancies and characteristics Motivation

COM-B, capacity, opportunity, motivation–behaviour; TDF, theoretical domains framework.

Illustrative quotes are from clinicians implementing the fan unless stated in the caption not implementing the fan. Further illustrative quotes are seen in S1, S2 and S3 Tables.

### Theme One Clinician knowledge and skills of fan implementation

#### Subthemes

*a) Explanation and scientific rationale*. Clinicians who implemented the fan reported working hard to present the fan as a credible intervention to patients and colleagues. Clinicians emphasized the details of potential mechanisms underpinning how the fan works supported by possible scientific rationale when encouraging patients to use the fan. Clinician’s implementation of the fan demonstrated a high level of competence and skills to deliver the intervention.

“I think the other side is possibly, and again it’s just the way I’ve developed it over time is I try and be very clear in the explanation when I’m talking about the fan is that there is a medical explanation into why it works and I’ll often draw parallels,….but to try and help patients understand potential mechanisms I talk to them a bit about how if they hit their hand with a hammer they shake it to try and relieve the pain and that works almost by the gate theory in terms of pain, so I talk a bit about how that may well also be the case in breathlessness, with the flow of air that you can stimulate the nerves within the airways and that can reduce the sensation of breathlessness. I try and provide almost a medical explanation to why something like that can work and tie it to another phenomenon that people are already familiar with.” Interview 4 *(doctor*, *respiratory medicine*, *hospital)*

*b) Complex intervention “part of toolbox”*. The fan was delivered consistent with the evidence base and clinicians considered this to be part of their skillset to manage breathlessness. Most clinicians delivered the fan as part of a complex intervention along with other breathlessness management techniques. It was considered an essential component of the toolbox.

“I suppose we deliver it as if it were a bit of a package I suppose. It’s not just about having the fan, it’s about what positions people get into to help and how they breathe as well, and again that’s what we’ve got from the X breathlessness service, the recovery breathing method that we teach that focuses on the 3 F’s, fans, thinking forward and focus on breathing out. It forms part of that whole package” Interview 11 *(respiratory nurse specialist*, *community)*

*c) Knowledge of fan research and opinion leaders*. Active reading of current research or conference attendance provided clinicians with current knowledge of the fan and improved their capability to deliver the fan in practice. Clinicians cited their connection to key fan researchers and opinion leaders as benefiting their clinical skills. This was fundamental to their belief in the intervention which drove clinicians to implement the fan in healthcare locations even if the fan was not imbedded within their services.

“I worked in X as registrar, in the breathlessness intervention service with X and the team and X was the researcher there at the time X was looking into fans, so it was kind of yes, it was very much imbedded in the culture there and I’ve never forgotten it since being something which has been very instrumental in my career.” Interview 8 *(palliative care consultant*, *hospice)*

### Theme two environmental constraints on fan use

#### a) Lack of access and funding for fan resources (respiratory vs palliative care setting)

Clinicians working in respiratory settings had no access to a supply of fans to directly provide patients and were limited to recommending that patients buy one. The only fans available were financed through charitable funding, which was restricted mainly to palliative care services.

“I know on the wards when they’ve been desperate to buy fans and they were struggling with the fan I don’t know where they got the funding in the end they were trying to just, we were hoping we’d be able to give them some fans, but if we give them our fans we won’t have enough for our patients and I don’t think they realised we were buying them out of our sort of patient equipment source charity pot.” Interview 6 *(physiotherapist*, *palliative care community)*

Clinicians felt strongly about the lack of resources and highlighted how it compromised their delivery of the fan. Patients were not able to experience the physical sensation and benefit of the intervention, limiting their buy in and could not be directly taught how to use it.

“If you had access there and then to actually show people so they can experience the good that would probably really improve any sort of scepticism from the patient point of view.” Interview 9 *(respiratory consultant*, *hospital)*

Further, clinicians could not be sure that patients would go and buy a fan, or, buy one with a sufficient flow-rate. Clinicians felt the device should be routinely funded and prescribed, in the same way that medications, inhalers and walking aids are supplied.

“I think in terms of provision, I don’t really understand why there wouldn’t be the provision of a fan, if you provide a stick for £2 that costs £2, why wouldn’t you provide a fan if it’s considered a clinical intervention why aren’t these things provided?” Interview1, disbelieving voice *(occupational therapist*, *palliative care*, *community)*

#### b) COVID-19 restrictions on fan use (acute vs community setting)

Clinicians reported that the COVID-19 pandemic guidelines banned nearly all fan use in acute healthcare settings for fear of spreading infection.

“..when national guidance came out about how we manage breathlessness in COVID patients there then was a strapline about avoiding the use of fans at that point so essentially they were taken away from what we could do both in the hospice when I was still working there and in the hospital…” Interview 7 *(palliative care consultant*, *hospital)*

However, fan use continued, with clinicians who visited patients in the community, particularly if patients were long-term fan users, as it was felt inappropriate to stop a patient from using the fan for breathlessness relief in their own home.

“… some of our patients we gave them (fans) to them years ago and they’re still using them because the thing that makes such a big difference to them and at this point we’re not going to say to them don’t use your fan you know just because of COVID being in the environment you can’t then take away someone’s almost lifeline of managing breathlessness that stops them calling 999 and admits to hospital every few weeks so you can’t really stop someone from using something that’s an effective tool, so we carry on giving them out but we warn patients that if they have any COVID symptoms or if they test positive then really they shouldn’t be using the fan in the presence of other people…” Interview 6 passionate voice *(physiotherapist*, *palliative care*, *community)*

#### c) Clinician education/awareness of fan (generalist vs specialist setting)

Clinicians perceived that outside of specialist respiratory services or palliative care the fan was rarely used, and the generalist setting lacked awareness of the fan, instead, favouring the routine use of oxygen or other pharmacological measures such as nebulisers and inhalers.

“And maybe getting the word out on the respiratory ward, a lot of the people who work on this ward, they haven’t actually managed respiratory patients before because they came in when it was COVID and it was a COVID ward, so I suppose doing some teaching just to say this is the option that’s out there for managing breathlessness, it’s not all about putting on nebulisers and oxygen.” Interview 11 *(respiratory nurse specialist*, *community)*

“Yeah I think people, certainly GPs, the information just doesn’t seem to reach them and some practice nurses the same because I’m sure they would you know they want to help this group, but I can’t imagine that there’s any other barrier really other than a lack of awareness, a lack of knowledge of how helpful it can be.” Interview 9 *(respiratory consultant*, *hospital)*

### Theme Three. Clinician beliefs about consequences of fan use

#### Subthemes

*a) Clinician beliefs about benefit from fan use*. Clinicians who delivered the fan to patients held strong beliefs about the benefits of fan use.

“So we had real faith, we knew it would work for them but you’re relying on the fact then that they would go to the shops for one or someone would go to the shops and get it… And the number of patients that said it does work like it’s a revelation to us, but we knew it would.” Interview 10 confident assured voice *(respiratory nurse specialist*, *hospital)*

The strength of the belief motivated clinicians to continue fan implementation despite their experience of negative views from patients and other clinicians of the fan as lacking credibility, coupled with the lack of resources.

“But I think there are probably some of my colleagues who have never recommended the fan at all and there’s people like me where anybody who has persistent breathlessness while I’m escalating their underlying disease process, they’ve got persistent and distressing and chronic breathlessness I’d recommend the fan.” Interview 4 *(doctor*, *respiratory medicine*, *hospital)*

*b) Clinician beliefs about infection risk (COVID-19) from fan use*. Most clinicians questioned whether fan use significantly increased the infection risk from droplet spread. They considered the possibility of transmission to be unlikely and if an appropriate risk assessment was followed then fan use was safe, particularly in the community where managing symptoms was prioritised over the risk of COVID-19.

“I mean I can’t imagine that there’s any evidence that the use of a handheld fan to help with breathlessness, increases anybody else’s risk of infection. It’s not going to generate enough to create aerosol, so the suggestion that droplets are going to spread further, I very much doubt it and given that the suggestions you should keep your windows open to increase your air exchange rates and reduce the amount of virus hanging around in the environment, you know having a bit of airflow in the room is probably increasing that as well.” Interview 4 Confident voice *(doctor*, *respiratory medicine*, *hospital)*

Some expressed concerns that fear of infection may hinder future patient fan use for breathlessness.

“I worry about the impact it’ll have going forward because it seems like we’ve thrown all the fans out with the bathwater and working to get them back in will take some doing. I think it’s hard to describe but I think we’re working quite risky environments but the real risk is far less than the real benefits the patients gaining and we’re losing sight on being able to risk assess appropriately.” Interview 5 Concerned voice *(Advanced Clinical Practitioner nurse*, *palliative care*, *hospice)*

*c) Patient and other clinician beliefs about fan credibility as intervention for breathlessness management*. Most clinicians reported that patients consistently viewed the fan as an implausible clinical intervention for breathlessness.

“It was so frustrating before because we’d keep advising it and advising it, but people didn’t have the belief to literally go and buy it.” Interview 10 Frustrated voice *(respiratory nurse specialist*, *hospital)*

Patients were perceived to believe that the fan was solely for cooling down in summer, was just “too simple” and could not possibly be an effective intervention or of value for breathlessness management.

“I give them a fan they’ll say, “but I’m not hot or if it’s in the middle of winter and its icy outside and they look at me and say well why am I going to use a fan in the middle of winter?” So they link using a fan with cooling themselves down on a hot summer’s day and not with breathlessness. But I have had some patients who have not maybe understood the instructions and literally I’ve said, “Have you used your fan?” and they’ve said, “Oh no it’s not been hot recently…” Interview 6 Frustrated voice *(physiotherapist*, *palliative care*, *community)*

This scepticism changed immediately it was demonstrated to them in practice and they felt the physical sensation from the device–raising concerns that patients might not try the fan if the clinician did not have one in clinic to demonstrate.

“I think we were giving them out, we gave them out because we found, from what I was told when I joined the service, that you give advice that someone should go and buy a fan and they don’t, you know because they think it’s either a bit of hocus-pocus or they don’t believe it works…..” Interview 6 *(physiotherapist*, *palliative care*, *community)*

Similarly, clinicians perceived that other clinicians who were more focused on a medical model and who were unaware of the developing evidence-base deemed the fan as a “soft” intervention.

“I know some of my colleagues are very sceptical that it [fan] seems a bit light touch a bit on that softer side isn’t it rather than a drug or an operation…” Interview 9 Sarcastic voice *(respiratory consultant*, *hospital)*

“If I went into work tomorrow with a box of fans, I know which members of staff I could give them to and they would start using them. I think on mass use, as a standard treatment you’re probably still years and years away, and they’ll be very late to doctors.” Interview 3 *(paramedic*, *emergency care*, *not implementing fan)*

## Discussion

We found three major themes in relation to clinician perceptions regarding implementation of the fan for breathlessness management; i) clinician knowledge and skills in fan implementation (explanation of mechanism and scientific rationale, complex intervention), ii) clinician beliefs about the consequences of fan use (benefit, low infection risk, patient scepticism), iii) environmental constraints on fan use (lack of resources, funding, awareness of the intervention and COVID 19 restrictions). These mapped well to capability (knowledge of evidence base, competence and skills), opportunity (resources, organisational culture including impact of COVID 19), and motivation (beliefs of benefit and low risk).

For clinicians who use the fan, delivery of the intervention is characterised by their knowledge and skills. Fan implementation involves clear explanation of the scientific rationale for use, coupled with varying strategies to promote patient belief and engagement. Clinician’s awareness of fan research evidence and knowledge of opinion leaders were cited as influential drivers in clinicians continuing professional development. This translated into embedded clinical practice such that fan implementation was a component of a complex intervention in keeping with current recommendations for breathlessness management [4,5].

One of the key environmental constraints was the lack of access to fans. Clinicians in respiratory settings experience inequity while those working in palliative care are fortunate to be able to access charitable funds for fans allowing provision and demonstration within the clinical encounter. This mirrors the Australian study of respiratory clinicians’ perspectives which found limited availability of fans in hospital environments [28]. Without a fan to use in clinic, the onus was placed on the patient to source a commercially available fan and start using it themselves. Models may vary and it may be difficult for the patient to know the best one to buy for breathlessness. A recent study of patient fan preferences [25] suggests that not all commercially available fans are as beneficial for breathlessness.

Despite presenting the fan as part of medical treatment, this approach did not address patients’ scepticism, or instil confidence in a device that was usually perceived for other purposes. This contrasted with interventions prescribed and funded by healthcare services—such as inhalers. However, it seems that current delivery strategies may not always be effective. Our finding that patients believe that a fan is too simple to be credible is consistent with a mixed-methods feasibility RCT [24] where such patient beliefs was a key concern for clinicians implementing the fan influencing uptake and long-term use [27].

The way a fan is introduced and taught to patients is important [26]. Clinicians’ explanations of chronic breathlessness and the words used in consultations may influence (positively or negatively) patient beliefs, expectations and understanding of their condition and how breathlessness should be managed [39–41]. However, breathlessness is often not discussed routinely by clinicians leading to persistent invisibility of this symptom in consultations [42–44]. Educational programmes for clinicians are of value to overcome this barrier [45]; inclusion of content about the fan may help clinicians introduce the intervention early in the context of a clear explanation of chronic breathlessness to patients. This could help clinicians to drive implementation in healthcare settings where there is a lack of awareness of the device and counter scepticism of a “soft” intervention which is defined by what it is not, “non-pharmacological” [26], an unhelpful term that does nothing to suggest the merits of the intervention.

### Strengths and limitations

Sampling for the interviews was limited to participants who had completed the survey which was unlikely to be representative of all eligible clinicians. Consistent with a non-representative sample most of those interviewed had connection or exposure to specialist breathlessness services or a fan research champion. Only one was not implementing the fan so our findings present a particularly positive view. However, clinicians who were implementing the fan freely reported other colleagues’ scepticism and lack of implementation and similarly of note so did the clinician not implementing fan. Although we did not reach our estimated sample size, data saturation was reached after 12 interviews consistent with our estimated information power with a limited topic [32,33]. FS acknowledges her clinical and research background helped frame the approach, however she maintained an open and critical mind throughout the research process. Two coders had no prior experience of fan implementation, but the dominant voices in the research team were from researchers with knowledge and experience of the fan.

### Implications for research and clinical practice

Access to resources and funding is a key issue that needs to be resolved if clinicians are to drive patient uptake of the device outside of palliative care and should be considered by all institutions and teams. Work to explore what fan explanations are most helpful and understandable to patients to promote effective engagement when delivering the fan would be useful.

Future studies are necessary to understand the decisions surrounding funding and what prevents or promotes allocation of finance for the fan in healthcare services, as well as the different possible funding models for equipment.

Research that simulates airflow to assess infection risk from fan use is indicated to inform review of COVID-19 guidance and determine the future of fan use in acute settings.

## Conclusion

Using behavioural change theory, we identified a number of motivators and de-motivators to implementing the fan in practice. Clinicians who implemented the fan were highly capable; characterised by knowledge and skills and influenced by opinion leaders in the field. Clinician beliefs in fan benefit and low infection risk were important motivators of implementation. Environmental barriers; lack of access to a supply of fans, patient disbelief in the intervention and COVID-19 constraints restricted opportunities and de-motivated clinicians implementing the fan in the UK. Many findings were similar to previous work from Australia, but the reliance on charity funding for fans in the UK created a specific inequity in provision.

## Supporting information

S1 File This is the S1 File showing a copy of the Participant Information Sheet and the informed consent form. (DOCX)

S2 File This is the S2 File showing a copy of the topic guide used in the clinician interviews. (DOCX)

S1 Table This is the S1 Table displaying further illustrative quotes from the participants in the study for Theme One, Clinician knowledge and skills of fan implementation. (DOCX)

S2 Table This is the S2 Table displaying further illustrative quotes from the participants in the study for Theme two, Environmental constraints on fan use. (DOCX)

S3 Table This is the S3 Table displaying further illustrative quotes from the participants in the study for Theme three, Clinician beliefs about consequences of fan use. (DOCX)
