# Supplementary material for: The handheld fan for chronic breathlessness: Clinicians’ experiences and views of implementation in clinical practice
Source: PLoS One. 2023 Nov 28;18(11):e0294748. doi: 10.1371/journal.pone.0294748 (PMC10684089; doi:10.1371/journal.pone.0294748)
Supplement: S1 File — (DOCX) [file pone.0294748.s001.docx]

**S1 Supporting Informtion**

**Participant Information Sheet and Informed consent form**

**Invitation to take part in a research study**

Thank you for completing the on-line survey about fan use or not in your clinical practice. We would like to invite you to take part in a follow up interview. To help you to decide if you would like to take part, we have devised this sheet. It explains why the research is being done, what you will be asked to do, and why we are inviting you to take part. Please take your time to read the following information; you might want to discuss it with your work colleagues, friends or family. Alternatively, you can contact the research team and ask them to explain anything that is not clear to you. The study is organised and run by researchers from the Wolfson Palliative Care Research Centre at the University of Hull. The contact details are provided at the end of this information sheet.

**What is the study about?**

As you know, we are investigating if and how the fan has been implemented or not in clinical practice with breathless patients. Patient research suggests that the fan is beneficial for the self-management of chronic breathlessness. We would like to find out more about your views and experience of using the fan or not with breathless patients, and what helped or stopped you from using the fan with patients.

We also want to ask you, your views about what you think are the key issues with implementing the fan in your clinical practice in the future, whether or not you used it previously with your patients.

We would also like to find out your thoughts about the current COVID 19 guidance not to use the fan and if, and how this is affecting your clinical practice with breathless patients who usually use the fan, but may have stopped using it because of the COVID 19 guidance.

**Why have I been invited to take part?**

You have been invited because you are a healthcare professional who is involved with the care of breathless patients.

**What will happen to me if I take part?**

If you are willing to take part in the study, please contact a member of the research team using the details at the end of this information sheet (by telephone, or email). The researchers will arrange an interview appointment which will be conducted by telephone at a time convenient to you. Interviews will be audio-recorded by the researcher to ensure that your answers are documented accurately. The interview will take about 30 minutes.

**Do I have to take part?**

No, it is up to you to decide whether or not you want to take part. The purpose of this information sheet is to give you information about the study and to help you understand what taking part will involve. After reading this information sheet you will have the opportunity to contact and discuss it further with the research team and ask any questions that you may have. If you decide to take part you are still free to withdraw at any time without giving a reason.

**What are the positives to be taken from participating?**

The information that we get from this study will help us answer important questions about the implementation of the fan for breathless patients and will allow us to understand what are the key barriers and facilitators to the uptake of the intervention in clinical practice. Your participation will ensure that the key issues which affect fan use in different clinical areas are highlighted. This may in the future help clinicians to implement the fan for breathlessness and improve patient access to the intervention.

**Are there any negatives to be considered should I decide to participate?**

We do not anticipate any disadvantage to you from taking part in this study other than taking your time. However, if you have any concerns, you can discuss these with the researcher or other clinical colleagues.

**Will my involvement be confidential?**

If you agree to be interviewed everything you say will be kept confidential and the information collected about you will be handled strictly in accordance with GDPR 2018. Transcripts will be anonymised, including places and names of third parties which might otherwise identify you.

Hull York Medical School will keep identifiable information about you (contact details and the audio recording) only until receipt and checking of the transcript. The only people who will have access to information that identifies you will be the researchers from Hull York Medical School who need to contact you about taking part in a study interview. To safeguard your rights, we will use the minimum personally-identifiable information possible and hold it for the shortest time possible. Identifiable information will not be shared with any third party.

The University of Hull is the Sponsor for this study and is responsible for looking after your information and using it properly.

If you withdraw from the study, we will keep the anonymised information about you that we have already obtained. You can find out more about how we use your information by contacting the Information Compliance Officer, University of Hull.

If you agree to take part in the study, your anonymised interview data may be provided to authorised researchers running other relevant research studies in this organisation and in other organisations. These organisations may be universities, NHS organisations or companies involved in health and care research in this country or abroad. Your information will only be used by organisations and researchers to conduct research in accordance with the [UK Policy Framework for Health and Social Care Research**.**](https://www.hra.nhs.uk/planning-and-improving-research/policies-standards-legislation/uk-policy-framework-health-social-care-research/) The information will not identify you and will not be combined with other information in a way that could identify you.

**How do I make a complaint?**

If you are not happy with your involvement in this study and feel unable to raise this directly with a member of the research team, or if you have any concerns about the way the researcher has carried out this study you may contact the research governance office at the University of Hull, email; [researchgovernance@hull.ac.uk](mailto:researchgovernance@hull.ac.uk)

**What will happen to the results of the research study?**

The results of this study will be published as journal articles and presented at conferences and public engagement events. A summary of the findings will be publically available on the Wolfson Palliative Care Research Centre website, <https://www.hyms.ac.uk/research/research-centres-and-groups/wolfson>

**Approvals**

All arrangements have been reviewed and approved by the Hull York Medical School Research Ethics Committee, University of Hull. The University of Hull has appropriate insurance and indemnity schemes in place relating to this research study.

**Who can I contact for further information?**

If you have any further questions about this research study, please do not hesitate to contact the Study Principal Investigator:

Dr Flavia Swan, Tel: 01482 463150 or 07794 297206 [flavia.swan@hyms.ac.uk](mailto:flavia.swan@hyms.ac.uk)

Thank you for taking the time to read this information sheet and if you do decide to take part, we very much appreciate your involvement.

Dr Flavia Swan and Prof Miriam Johnson

Wolfson Palliative Care Research Centre, Hull York Medical School

**Clinical staff Informed Consent Form**

**Clinical staff interviews: Fan implementation study**

**Name of lead researcher: Flavia Swan**

| **Please note: Consent will be taken verbally – the interviewer will ask and record your consent to each point below prior to starting the interview.** |
| --- |
| I confirm that I have read the participant information sheet (version 1.1) for the above study. I have had the opportunity to consider the information, ask questions and discuss this study. I have received satisfactory answers to all of my questions. |
| I understand that my participation is voluntary and I am free to withdraw from the study at any time and without having to give a reason. |
| I understand that the telephone interview will be audio-recorded by the researcher to ensure that my views are documented accurately. |
| I agree that anonymous quotations from my interview can be used in presentations or publications arising from this project. |
| I agree that anonymised data can be used by authorised researchers working on similar studies |
| I understand that data collected during this study may be looked at by responsible individuals from the research team, where it is relevant to my taking part in this research. I give permission for these individuals to have access to my data. |
| I agree to take part in an interview for this study. |

**Clinical staff Signature**......................................................................................................

**Print Name**....................................................................**Date**...............................................

**Investigator Signature**.............................................................................................................

**Print Name**...........................................**........................Date**....................................................
