# Supplementary material for: The handheld fan for chronic breathlessness: Clinicians’ experiences and views of implementation in clinical practice
Source: PLoS One. 2023 Nov 28;18(11):e0294748. doi: 10.1371/journal.pone.0294748 (PMC10684089; doi:10.1371/journal.pone.0294748)
Supplement: S2 File — (DOCX) [file pone.0294748.s002.docx]

**S2 Supporting Information**

**Topic guide for clinical staff interviews**

Introduce researcher

Check if any questions and record verbal consent

Check happy to be recorded and knows that recording has started

**For those that use the fan**

OPEN QUESTION:

- Tell me about your clinical experience with the handheld fan?
- How did you find using the handheld fan in clinical practice?

What influences your use of the fan in clinical practice?

**Probes positive**

Relevant domains from TDF will be used to structure probes if necessary: (*knowledge, skills, social/professional role and identity, beliefs about capabilities, beliefs about consequences, environmental context and resources, social influences)*.

OPEN QUESTION:

- Tell me about your current experience and views on using the fan with breathless patients?
- How do you find using the fan with breathless patients?

**Probes** Policy, guidelines, research evidence, beliefs, patient view

Any other thoughts or is there anything about (x) we have missed that you want to tell us about?

**For all**

What stopped you using or made it difficult for you to use the fan in clinical practice?

**Probes negative**

Relevant domains from TDF will be used to structure probes if necessary: (*knowledge, skills, social/professional role and identity, beliefs about capabilities, beliefs about consequences, environmental context and resources, social influences)*.

**For all**

What would help you to use the fan in clinical practice in the future?

**Probes positive**

1. Perceived benefits and advantage– patient, carers, clinicians
2. Clear written protocol including fan use and importance of how fan is delivered
3. Regular source of fans
4. Adequate resources; fans, spare batteries and information sheets for patient education
5. Consistent fan use across clinical team /department/healthcare services
6. Clinical team presented with fan evidence/rationale
7. Adequate finance
8. Presence of a research champion clinician who champions fan cause and is credible

How do experiences (in different domains) fit together, e.g. how does knowledge, social influences, and resources intersect?

Any other thoughts or is there anything about (x) we have missed that you want to tell us about?
