# Supplementary material for: The handheld fan for chronic breathlessness: Clinicians’ experiences and views of implementation in clinical practice
Source: PLoS One. 2023 Nov 28;18(11):e0294748. doi: 10.1371/journal.pone.0294748 (PMC10684089; doi:10.1371/journal.pone.0294748)
Supplement: S1 Table — (DOCX) [file pone.0294748.s003.docx]

**S3 Table**

**Theme One, Clinician knowledge and skills of fan implementation illustrative quotes**

| **Theme** | **Participant Quotes** |
| --- | --- |
| **Theme 1 Clinician knowledge and skills of fan implementation** | |
| **a) Sub-theme**  **Explanation and scientific rationale** | “I have found that explaining, for some people, explaining the kind of physiological reasoning behind it and that actually there is some sense behind it and there is some science behind it, that sometimes can make it more acceptable to people. But yeah because it’s not a medical thing or a medical device or a tab or something you are giving people there is that perception.” Interview 2 *(physiotherapist, respiratory, community)*  “You need to give them that explanation about you know it's to do with receptors on your face that you’re stimulating the circulation of air and you know how it works I usually do it a little bit like that and give them some scientific rationale if you like.” Interview *7 (palliative care consultant, hospital)*  “I’ll say have you got a fan, and they’ll say ooh yes, and I’ll say where is it and it’s in a drawer just gathering dust and I think if you haven’t actually explained why it works, what the research is and actually got them to try it out while they’re with you, they don’t always even take the easy option if you just give it, it’s not something you can literally just post out and expect them to use it, you do have to have an explanation alongside it and although we’ve got a leaflet often a physical face-to-face verbal explanation seems to work best.” Interview 6 *(physiotherapist, palliative care, community)* |
| **b) Subtheme Complex intervention** | “I would say that I consider it [fan] as one of my key tools in my toolbox for managing breathlessness.” Interview 8 *(palliative care consultant, hospice)*  “We talk about fan therapy a lot with our patients, so we cover we provide a breathlessness management education session as part of a pulmonary rehab. And we talk about fan therapy as part of that programme…” Interview 2 *(physiotherapist, respiratory, community)*  “…but we’ll also look at sort of encouraging people to have the tools to self-manage their condition and be in control so they can live their life better, so obviously management of breathlessness is a big part of that and fan therapy is a part of that as well as breathing techniques.” Interview *11 (respiratory nurse specialist, community)* |
| **Subtheme: c) Clinician knowledge of fan research and research champions** | “I’ve picked up quite a bit of knowledge really from people like X when she talks at conferences and I do think conferences are a really good way of sharing education and X did a brilliant talk at the X about how to manage breathlessness and I'm sure everyone in that room is much better now at showing people how to use a fan.” Interview 9 *(respiratory consultant, hospital)*  “I stumbled across the X breathlessness intervention service and started reading their work. I got their book and obviously within that it talks about, and I was aware they do a conference about managing breathlessness, but I couldn’t afford to go or to travel all the way down there. So, I arranged to bring them up here, so we’ve had two conferences they’ve put on up here to get the word out about managing breathlessness up here to get the word out.” Interview 11 *(respiratory nurse specialist, community)*  “I don’t know whether it is motivational interviewing but the technique where you get people to actually recognise their own buy in. I definitely learnt that from X, I remember her saying, do you ever get benefit from winding the car window down or opening a window in the house and it’s that sort of thing isn’t it, that lead in. You’ve already worked out that this helps…” Interview 9 *(respiratory consultant, hospital)* |
