# Supplementary material for: The handheld fan for chronic breathlessness: Clinicians’ experiences and views of implementation in clinical practice
Source: PLoS One. 2023 Nov 28;18(11):e0294748. doi: 10.1371/journal.pone.0294748 (PMC10684089; doi:10.1371/journal.pone.0294748)
Supplement: S2 Table — (DOCX) [file pone.0294748.s004.docx]

**S4 Table**

**Theme two,** **Environmental constraints on fan use illustrative quotes**

| **Theme 2** **Environmental constraints on fan use** | |
| --- | --- |
| **a) Subtheme:**  **Lack of access and funding for fan resources (respiratory Vs palliative care setting)** | “…We have a lot of conversation on the hospital wards about you know managing breathlessness but what we don’t have is a handheld fan to actually give people whereas you know you can prescribe inhalers you can prescribe everything else and that always seems a bit of a shame that you can’t at the time you're having this discussion actually give people what you're saying is helpful….” Interview 9 *(respiratory consultant, hospital)*  “We’ve often said as a team that we think it would help to have a small stash that we could give out to patients, and I think we have looked at that charitable funds and things for those. But we’ve, yeah, it tends to be actually accessing them. If people are online they can order them on the internet, but the people that are sort of, perhaps can’t get to the shops and aren’t internet savvy having access to getting one is sometimes the problem. We have suggested as an idea, but ultimately if they don’t have one and can’t get one it’s not very helpful.” Interview 2 *(physiotherapist, respiratory, community)*  “No, we don’t, we have no funding for stock. So historically I know the palliative care team got some money in the hospital, so we could steal them I suppose. We used their stock for a time, but that’s all gone now. So yeah, it relies on good gesture I suppose from patients. Our colleagues from the heart failure team, they’ve recently been given some money from a patient so they’re going to invest that into a stock of fans, but we haven’t been given any more so we can’t do it.” Interview 11 *(respiratory nurse specialist, community)*  “I think it would be really helpful to have something (fan) that has been identified as valid and effective. So therefore, I think that would make it easier to have it put into the mainstream financial requirements. I think you could then get it prescribed.” Interview 13 *(palliative care nurse, hospital)* |
| **b) Subtheme:**  **COVID-19 restrictions on fan use (acute Vs community setting)** | “So I know, I have spent a little bit of time working in an acute hospital at the start of COVID and obviously fans, we were advised not to use fans uhh with patients, and I have had one community patient who got admitted to hospital and then self-discharged herself because she found it so difficult not being able to have a window open or have a fan on.” Interview 2 *(physiotherapist, respiratory, community)*  “I was redeployed 3 times during COVID so I did get to see the patients on the wards and there was no mention of fan therapy for those patients that were breathless at all…” Interview 11 *(respiratory nurse specialist, community)*  “We have to tell our patients now with the handheld fan to save it and try it when they get home.” Interview 10 *(respiratory nurse specialist, hospital)* |
| **c) Sub-theme: Clinicians awareness of fan (generalist Vs specialist setting)** | “physiotherapists in our patch are very knowledgeable so if they do come to rehabilitation they will have some teaching around fans and things but even that can be quite hit and miss depending on who they see you know it's not a formal part of their education, part of rehabilitation, whereas perhaps it should be” Interview 9 *(respiratory consultant, hospital)*  “I worked it was quite a specialist respiratory ward, so all the nursing staff and healthcare assistants were sort of quite umm knowledgeable if you like, about respiratory conditions and knew the benefit of the fan. Whereas if they had sort of gone to a general medicine ward or you know perhaps been on an outlying ward then yeah it probably wouldn’t have been so available.” Interview 2 *(physiotherapist, respiratory, community)*  “As far as I was aware, they hadn’t actually been implemented, I’ve never seen one. I’ve not had training on how to implement one.” Interview 3 *(paramedic, emergency care, not implementing fan)* |
