# Supplementary material for: The handheld fan for chronic breathlessness: Clinicians’ experiences and views of implementation in clinical practice
Source: PLoS One. 2023 Nov 28;18(11):e0294748. doi: 10.1371/journal.pone.0294748 (PMC10684089; doi:10.1371/journal.pone.0294748)
Supplement: S3 Table — (DOCX) [file pone.0294748.s005.docx]

**S5 Table**

**Theme three, Clinician beliefs about consequences of fan use illustrative quotes**

| **Theme 3**  **Clinician beliefs about consequences of fan use** | |
| --- | --- |
| **Subthemes**  **a) Clinician beliefs about benefit from fan use “know it will work”** | “No, I am already a big advocate of the fan, I have recommended several times in every clinic to patients with persistent breathless, and I have to say the majority do agree to give it a go and the ones that do tend to find it useful.” Interview 4 *(doctor, respiratory medicine, hospital)*  “I’m hoping, I would really hope that it wouldn’t reduce fan use in people who aren’t COVID positive because that would be such a shame because it’s such an amazing tool.” Interview 6 *(physiotherapist, palliative care, community)*  “We absolutely advise use of a handheld fan and it is surprising how many people have never had that information even though these people have been in and out of hospital multiple times you know have had multiple healthcare contact along the way but it's taken someone to come up to what really is a tertiary referral specialist clinic to actually talk about really quite straight forward low cost management and our anecdotal evidence, we’ve not collected any data but it is just that people find it so helpful.” Interview 9 *(respiratory consultant, hospital)*  “I think it was much more of a fact that it was being used routinely within community practice so even though there wasn’t, there has been patients that have reported evidence of it that kind of initiated the interest. It was much more of the team believing in it as well, so people buying in to it, seeing evidence and seeing it benefit.” Interview 13 *(palliative care nurse, hospital)* |
| **b) Clinician beliefs about low infection risk (covid-19) from fan use.** | “I think we’ve lost our heads slightly around a lot of things in terms of balancing the actual real risk and the benefit for patients to the point where I’ve known occasions where patients have been dependant on their fan use and it’s been withdrawn, even though they don’t have COVID and there’s no COVID in the building. It just doesn’t make sense that that would happen. So, yeah I think that’s a mix.” Interview 5 *(Advanced Clinical Practitioner nurse, palliative care, hospice)*  “I don't know the evidence behind it but personally, I think the fan can be such a useful component of somebody symptom control and can relieve distress. I certainly have been making decisions on a case-by-case basis rather than just deciding that fans shouldn't be used.” Interview 8 *(palliative care consultant, hospice)*  “I think it depends where you are. I think if you were in an environment with AGPs and bits and pieces and high-risk areas where COVID is very positive then I think we need to be very careful. But I think in the majority of areas as long as the patient is using it in the correct way, on their face and not wafting it around willy-nilly then I think it’s perfectly safe.” Interview 13 (nurse, palliative care, hospital) |
| **c) Subtheme: Patient and other clinician beliefs about fan plausibility as intervention for breathlessness management**  **“too simple” and “soft”** | “So, I think because it hasn’t got the kudos as say a tablet which you get prescribed and get delivered and is documented and is given on a once daily basis or 3 times daily basis. You know you have a prescription; you know is there something around trying to identify some kind of prescription that would be helpful, and then evaluation. And actually, having a really clear care plan of action from the use of the fan as well.” Interview 13 *(palliative care nurse, hospital)*  “I'm more of a believer in it than my colleagues in the respiratory service and then I understand yeah the patients in the hospice's I find that probably I'm the one who will say, “Well have they got the fan?”, and someone else say “Oh yeah someone else mentioned that then we'll get it.” But yeah it's I feel like it hasn’t become as embedded as I hoped it might do, other people don't believe in it as much as I do.” Interview 8 *(palliative care consultant, hospital)* |
